# Effects of stereospecific positioning of fatty acids in triacylglycerol structures in native and randomized fats: a review of their nutritional implications

**DOI:** 10.1186/1743-7075-4-16

**Published:** 2007-07-12

**Authors:** Tilakavati Karupaiah, Kalyana Sundram

**Affiliations:** 1 Department of Nutrition & Dietetics, Faculty of Allied Health Sciences, National University of Malaysia, Jalan Raja Muda Abdul Aziz, Kuala Lumpur 50300, Malaysia; 2 Malaysian Palm Oil Council (MPOC), 2nd Floor Wisma Sawit, Lot 6, SS6 Jalan Perbandaran, 47301 Kelana Jaya, Selangor, Malaysia

## Abstract

Most studies on lipid lowering diets have focused on the total content of saturated, polyunsaturated and monounsaturated fatty acids. However, the distribution of these fatty acids on the triacylglycerol (TAG) molecule and the molecular TAG species generated by this stereospecificity are characteristic for various native dietary TAGs. Fat randomization or interesterification is a process involving the positional redistribution of fatty acids, which leads to the generation of new TAG molecular species. A comparison between native and randomized TAGs is the subject of this review with regards to the role of stereospecificity of fatty acids in metabolic processing and effects on fasting lipids and postprandial lipemia. The positioning of unsaturated versus saturated fatty acids in the *sn*-2 position of TAGs indicate differences in early metabolic processing and postprandial clearance, which may explain modulatory effects on atherogenecity and thrombogenecity. Both human and animal studies are discussed with implications for human health.

## Background

For almost two decades, studies on lipid lowering diets examined the role of saturated, monounsaturated and polyunsaturated fatty acids in affecting plasma low density lipoprotein-cholesterol (LDL-C) concentrations, since foam cell formation triggered by elevated LDL-C lays the foundation for atherosclerotic plaques. This landscape changed in the 1990s, when serum triacylglycerol (TAG) was identified as an independent cardiovascular risk factor, and triacylglycerol-rich lipoproteins (TRL) became implicated in the development of atherosclerosis [1,2]. The progression of coronary atherosclerosis amongst non-diabetics in the Monitored Atherosclerosis Regression Study (MARS) was related to TRL levels, whilst angiographic severity of coronary artery disease (CAD) in another non-diabetic population was reported to be greater with higher plasma TAG levels [3-5]. In type 2 diabetes, angiographic severity of CAD was positively related to the numbers of circulating TRL particles in plasma, and this relationship was stronger in women than men, and independent of high density lipoprotein (HDL) and LDL [6]. In non-diabetics with moderate hypertriglyceridemia, about 75% of plasma TAG increase was attributed to the increased number of TRLs, with small particle sizes (Sf 12 to 60).

The metabolism of TRLs and their effects on remodeling LDL and HDL during reverse cholesterol transport plays a major role in the later stages of atherothrombotic progression [7]. Elevated TAG, resulting from a fat-rich meal, triggers a chain of metabolic events that reduces HDL-C, promotes formation of small dense LDL particles, and activates factor VII (FVII) [8-10]. The size and lipid composition of TRL particles in chylomicrons may be involved in pro-inflammatory atherogenic processes. The induction of endothelial dysfunction, as well as a prothrombic state, contributes to cardiovascular dysfunction [11]. The small size of LDL and its concomitant increased density and particle number, is also a recognized atherogenic stimulus. Small dense LDL develops through interactions with TRLs, particularly large very low density lipoprotein (VLDL) in the postprandial phase [12].

The prime interest of this review is to examine effects on lipids and lipoproteins associated with the positional distribution of dietary fatty acids, esterified to carbon atoms forming the glycerol backbone of TAG structure. Comparisons include both native TAGs and TAGs formulated in a random or purposeful fashion. Alterations to the TAG structure are manifested through randomization of native fats, and the terms "randomization" or "interesterification" are used synonymously to designate the process. Alternately, specific fatty acids can be built into TAG molecules and such synthetic fats are referred to as "structured fats". To a large extent TAG structure is important in food processing since this influences melting characteristics and crystallization properties of individual fatty acids making up the TAG molecular species. With the Institute of Medicine [13] recognition that *trans *fatty acids arising from hydrogenation are associated with increased risk for cardiovascular disease, randomized fats are viewed as a possible alternate by the food industry to fill the void created by the exit of hydrogenated fats.

The metabolic effects of randomized fats, has been sparsely studied in humans, and literature to date is confined mostly to animal studies [14-16]. Early animal models of piglets, dogs, rats and hamsters utilized semi synthetic fats to elucidate the behavior of specific fatty acids in relation to hypercholesterolemia and the progression of atherosclerotic lesions [17,18]. In contrast, increasing citations on the use of structured lipids in catabolically stressed hospital patients is now apparent, but such literature is not within the scope of this review. Some recent human postprandial studies [19-22] have probed TAG structural influences on digestion, absorption and transport of TAG molecular species rising from C16:0-rich, C18:1-rich and C18:0-rich fats.

In the subsequent discussion, abbreviations are used to represent TAG species. For example, POO represents 1-palmitoyl-2-oleyl-3-oleyl-*sn*-glycerol and the following symbols are used to represent the fatty acids discussed in this review: P = palmitic; O = oleic; S = stearic; D = dodecanoic or lauric; C = capric; M = myristic; L = linoleic; Ln = linolenic; B = butyric.

### Stereospecificity of native dietary TAGs

#### Structure and function

In the native TAG molecule, fatty acids are esterified to three stereospecific positions on the glycerol backbone. The positions occupied by these fatty acids are numbered relative to their stereospecificity or stereospecific numbering (*sn*) as *sn*-1, *sn*-2 and *sn*-3. The orientation of TAG structure stereospecificity is as follows: if the fatty acid esterified to the middle carbon of the glycerol backbone is considered to the left (on the plane of the page), then the top carbon is numbered *sn*-1, the bottom carbon is numbered *sn*-3 (below or behind the plane of the page) and the middle carbon is numbered subsequently as *sn*-2 [23]. The type of fatty acid and its stereospecificity in TAG molecular species, largely determines the physical behavior of dietary fats as a whole in food products [24,25]. Quality specifications such as 'mouth feel' in chocolate or ice-cream, and the 'lightness' of pastry are dependent on melting point and crystallisation properties of fats. The melting point of chocolate, just below body temperature, can be attributed to C18:0 and C16:0 exclusively at *sn*-1/3 and C18:1 at the *sn*-2 positions.

#### Characteristic distribution of TAG species

The stereospecificity of fatty acids in TAGs are characteristic for native oils and fats as indicated in Table 1[26-29]. TAG molecules making up adipose tissue of animals, largely have a saturated fatty acid (SFA) at the *sn*-1 position and an unsaturated fatty acid at the *sn*-2 position [24,27]. For instance, in beef tallow C16:0 is at *sn*-1 position and C18:1 is at *sn*-2 position as seen in POO, POP and POS TAGs but vary in the fatty acids located at *sn*-3. Contrary to this, in butter fat, C16:0 is not exclusive to *sn*-1 but occupies *sn*-2 in two-thirds of TAG species as seen for PPB, PPC and PPO TAGs. In lard, C16:0 is located exclusively at the *sn*-2 position, with an unsaturated fatty acid at *sn*-3 but the fatty acid occupying the *sn*-1 position is highly variable, as in SPO, OPL and OPO TAGs [28].

**Table 1:** Stereospecificity of dominant TAG molecular species in natural fats and oils

	***TAG species***		
***Vegetable Oil***			

Almond oil	OOO	OLO	OLL
Canola oil	OOO	LOO	OOLn
Cocoa butter	POS	SOS	POP
Coconut oil	DDD	CDD	CDM
Corn oil	LLL	LOL	LLP
Cottonseed oil	PLL	POL	LLL
Olive oil	OOO	OOP	OLO
Palm kernel oil	DDD	MOD	ODO
Palm oil	POP	POO	POL
Peanut oil	OOL	POL	OLL
Safflower oil	LLL	LLO	LLP
Soybean oil	LLL	LLO	LLP
Sunflower oil	LLL	OLL	LOO
Walnut oil	LLL	OLL	PLL

***Animal Fat***			

Butter	PPB	PPC	POP
Egg fat	POO	PLO	POS
Lard	SPO	OPL	OPO
Tallow	POO	POP	POS

For fatty acid abbreviations in TAG species read as: P = palmitic; O = oleic; S = stearate; D = dodecanoic or lauric; C = capric; M = myristic; L = linoleic; Ln = linolenic; B = butyric.

In most vegetables oils, either C18:1 or C18:2 are exclusively at the *sn*-2 position in TAG species like OOO, LLL, POL and LLO [24,29]. Linolenic acid (C18:3) occurs less commonly, but when present, is at the *sn*-3 position as seen for OOLn in canola oil. SFAs in vegetable oils such as palm oil and cocoa butter, occur primarily at the *sn*-1 position as in POL, POO, POS, SOS, POP and PLL, rarely at the *sn*-3 position such as POP, LLP and OOP, but to a lesser extent at the *sn*-2 position. Contrary to this trend, in coconut oil most SFAs ≤ 14 carbon atoms are equitably distributed in all three stereospecific positions as seen in DDD, CDD and CDM species (M = myristic; D = dodecanoic or lauric acid; C = capric), with C12:0 characteristically occupying the *sn*-2 position. Oleic acid is prevalent in TAG species of animal or plant origin, commonly at the *sn*-2 position as observed in olive oil, beef tallow, cocoa butter, palm oil, peanut oil and canola oil but exclusive to the *sn*-1 and *sn*-3 positions only in lard [24,29].

The difference between bovine and human milk fat lies in the amount of SFA present in the *sn*-2 position. In bovine milk, C16:0 and C18:0 are equitably distributed between *sn*-1/3 (34% and 10%) and *sn*-2 positions (32% and 10%). Human milk has more C16:0 (58%) and less C18:0 (3%) occupying the *sn*-2 position, in contrast to their amounts in the *sn*-1/3 positions (16% and 15% respectively). In addition to various natural dietary fats possessing characteristic TAG molecular species, the presence of asymmetrical species in vegetable oils has been noted. The development of advanced techniques for stereospecific analysis of TAG fatty acids proved the existence of asymmetry between *sn*-1 and *sn*-3 positions [30,31]. Asymmetrical fatty acids in these *sn*-1/3 stereochemical positions are found in most vegetable oils, and do not exhibit random behavior.

### Postprandial fate of native dietary TAGs

#### Hydrolysis in the duodenum

The stereospecificity and chain lengths of fatty acids, at the *sn*-1, *sn*-2 and *sn*-3 positions in TAG species, determine the metabolic fate of dietary fat during digestion and absorption [26,32]. The enzymatic hydrolysis of dietary TAGs is a major activity of digestion, largely occurring in the duodenum. Preferential hydrolysis by pancreatic and lipoprotein lipases target the fatty acids in the *sn*-1 and *sn*-3 positions resulting in free fatty acids (FFAs) and *sn*-2 monoacylglycerols [33,34].

It has been suggested, that pancreatic lipase shows greater affinity for ester bonds in the *sn*-1 position, compared to the *sn*-3 position [35]. Ultimately however, all fatty acids in the *sn*-1/3 positions of TAGs are hydrolyzed during digestion, in contrast to only 22% of fatty acids in the *sn*-2 position [36]. Mu and Hoy [37] estimate an approximate 75% conservation of fatty acids in the *sn*-2 position, despite acyl migration to the *sn*-1/3 positions. They suggest a major factor promoting this conservation is the regiospecificity of pancreatic lipase for fatty acids in *sn*-1/3 positions as well as the chain length of these fatty acids. Long-chain fatty acids (LCFAs) that contain more than 12 carbons, and short- and medium-chain fatty acids originating from the *sn*-1 and *sn*-3 positions, undergo different absorption pathways, as will the *sn*-2 monoacylglycerols [34]. LCFAs require a protein-mediated process, whilst *sn*-2 monoacylglycerols are absorbed by passive diffusion [38]. *In vitro *studies with adipocytes suggest the possibility that fatty acid transport proteins may have different affinities for different fatty acids, depending on chain length [39,40].

#### Changes in chylomicron

Upon entry into the enterocyte, the major site of metabolism of fatty acids will be the endoplasmic reticulum where TAG structures are reassembled and packaged into chylomicrons. There is substantial preservation of the fatty acid at the *sn*-2 position of the ingested TAG in chylomicrons after digestion [34,41]. Stereospecific structure of the ingested TAG is also largely preserved in chylomicrons [20]. Reassimilation of absorbed *sn*-2 monoacylglycerides and FFAs occurs via the monoacylglyceride pathway, or alternately by the α-glycerophosphate pathway, if the supply of FFAs is more than the monoacylglycerides [42,43]. The *sn*-2 monoacylglycerols, being absorbed intact, will serve as a primary backbone for gut or liver phospholipid synthesis in excessive FFA environments. Otherwise, *sn*-2 monoacylglycerols are rapidly esterified with FFAs into TAGs, which are then incorporated into lymph chylomicrons.

The metabolic fate of fatty acids released from *sn*-1 and *sn*-3 positions are different from the *sn*-2 monoacylglycerols. Fatty acids from the *sn*-1 and *sn*-3 positions, with chain lengths greater than 12 carbon atoms (long chain fatty acids or LCFAs), are reassembled into new TAG structures via the phosphatidic acid pathway, which is slower, and which possibly lowers secretion into the circulation [34]. Additionally, LCFAs have low coefficients of absorption because their melting points are higher than body temperature, and can form insoluble soaps with calcium. Despite the regiospecificity for fatty acids in the *sn*-1/3 positions, initial low activity of pancreatic lipase towards *n*-3 fatty acids specific to the *sn*-3 position, causes a lag phase in the supply of *sn*-2 monoacylglycerols, which eventually delays formation of TAG in intestinal mucosal cells [37]. Salmon and seal oils, despite sharing a similar FAC, differ in the stereospecific distribution of s*n*-3 PUFAs. In salmon oil the *sn*-3 FAs are mostly located in the *sn*-2 position but in seal oil it is preferentially located at the *sn*-3 position. Pancreatic lipase activity towards C20:5n-3 and C22:6n-3 is lower compared to other FAs when located at *sn*-3 position [44]. Ikeda et al. [45], in comparing TAGs containing C20:5n-3 and C22:6n-3 to triolein, found slower hydrolysis. By using mesenteric lymph collection in rats, lymph transport following salmon oil was higher in the first 8 h compared to seal oil, indicating a faster hydrolysis of salmon oil by pancreatic lipase [46]. However, over 24 h no difference in accumulated transport was seen between the oils. Asymmetry may perhaps explain the initial low activity of pancreatic lipase for LCFA in the *sn*-3 position [47,48].

In contrast, fatty acids of short (C4-C6) and medium (C8-C10) chain fatty acids (SCFAs and MCFAs) solubilize in intestinal fluids and are absorbed directly into the portal system. Once in portal circulation the SCFAs and MCFAs will form complexes with albumin and are carried to the liver for oxidation.

#### TAG clearance

The amount of TAGs carried by chylomicrons during the absorptive stage will vary depending upon the amount of dietary TAG consumed, and dietary meal constituents. Following fat absorption, chylomicrons are released into intestinal lymphatics, enter circulation via the thoracic duct and move to capillary surfaces of target organs (liver, heart, etc) or tissue (adipose, skeletal), where TAG disassembly takes place. The norm after a meal is that chylomicron breakdown will be directed towards adipose tissue through the activation of adipose tissue lipoprotein lipase (LpL). This pathway is triggered by the elevation of plasma insulin levels which increase concurrent to a meal. In the fasting state, the hormonal balance favors skeletal tissues by the activation of muscle LpL. The triple functionality of LpL in relation to processing chylomicrons is now well-established [49]. Through a process called margination, LpL first attaches chylomicrons to glucosaminoglycans lining the endothelial wall, lipolyzes the chylomicron TAGs, and then mediates their binding to specific lipoprotein receptor protein for liver uptake [50,51]. LpL exhibits regiospecificity for fatty acids in the *sn*-1/3 positions similar to pancreatic lipase [52]. Initially, susceptibility to LpL hydrolysis of chylomicron TAGS is said to favor the *sn*-1 position and results in diacylglycerols comprising *sn*-2/3 FAs [53,54].

A high LpL activity is associated with a faster chylomicron TAG clearance, and similarly with low LpL activity, chylomicron clearance will be slowed down [51]. The rate of hydrolysis is determined by the number of active LpL molecules in contact with the TAG molecule, as well as the removal of liberated fatty acids from the capillary microenvironment. If free fatty acids are not readily removed, then capillary concentrations will rise rapidly, and LpL activity will become product inhibited [55]. Prematurely, chylomicrons and LpL will detach from the endothelial surface (of adipocytes), leading to the formation of triacylglycerol-rich chylomicron remnants or TRL remnants [56,57]. The rate of hydrolysis is not determined by LpL itself, as LpL mass is present normally in excess of need [58]. Instead the hydrolysis rate is controlled by acylation stimulating protein (ASP) and insulin, via fatty acid trapping into adipocytes. In healthy humans, half the fatty acids released from chylomicrons enter adipocytes, and the other half enters the circulation [59].

The entry of TAGs into the lymphatics via chylomicrons also provides primary substrates for the 'core pathway' of the reverse cholesterol transport. The core pathway is the means by which cholesteryl esters (CE) in HDL are selectively transferred to chylomicrons, in exchange for TAGs, aided by cholesteryl ester transfer protein (CETP) [60]. As delipidation continues, there will be changes in density and size of both HDL and chylomicrons. Chylomicron particles become progressively smaller as TAG is depleted, and will be converted into chylomicron remnants [61]. Chylomicron remnant particles carrying CE are removed by the liver, through the apo E receptor [62-64]. The core pathway represents the metabolic route for postprandial lipemia. Chylomicron particles and their remnants are hypothesized as the major contributors to atherosclerosis, as excessive postprandial lipemia is associated with increased risk for myocardial infarction (MI) and stroke [51].

### Altering stereospecificity of fatty acids

#### Modification of fats

Interesterification is currently viewed as an alternate process to the partial hydrogenation of oils and fats. The process involves randomization among all three stereospecific positions, of fatty acids in native edible oils and fats by either enzymatic or chemical catalysis, at low temperatures. The positional distribution of fatty acids on the glycerol backbone is altered either through fatty acids switching positions within a TAG molecule, or between TAGs. If interesterification involves TAG species within the same dietary fat, the fatty acid composition remains the same. Sometimes a solid fat is interesterified with liquid oil, and in this case fatty acid composition of the interesterified product will differ.

**Figure 1:**
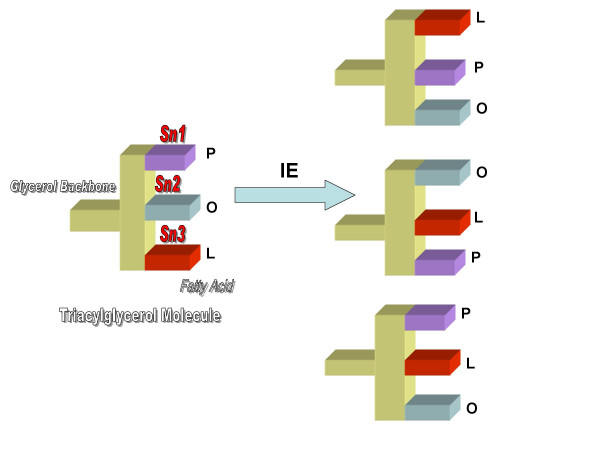
**Triacylglycerol molecule structure before and after interesterification**. An example of a triacylglycerol molecule (POL) that occurs in oils and fats is depicted in which palmitic (P), oleic (O) and linoleic (L) fatty acids occupy the Sn1, 2 and 3 positions respectively. Following interesterification (IE) by either chemical or enzymatic methods, these fatty acids are rearranged and take new forms, which would include LPO (linoleic-palmitic-oleic), OLP (oleic-linoleic-palmitic) and PLO (palmitic-linoleic-oleic) among the various permutations that are possible. Since natural oils and fats are made up of a variety of triacylglycerol molecules, interesterification will result in a large number of new triacylglycerol molecules.

Randomization allows component fatty acids to be esterified equally [one-third content] to all three glycerol carbon atoms (Figure 1), whereas the distribution is specific and unequal in naturally occurring fats. For example, McGandy et al. [65] devised semi synthetic fats, in an attempt to elucidate the specificity of cholesterol-raising properties of dietary fatty acids. Their fats were developed by interesterification of naturally occurring fats with trilaurin, trimyristin, tripalmitin, and partially hydrogenated soybean oil (providing 85% of C18:0). Interesterification thus changes the amount of fatty acids located at the *sn*-2 position of TAG molecules. A summary of *sn*-2 fatty acid comparisons, between some native oils and fats and their randomized versions, is presented in Table 2.

**Table 2:** Approximate *sn*-2 fatty acid composition of native *vs *randomized fats [mol%]

		**native**		**Randomized**	
		
**Type of fat**	**Fatty acid**	**Total FAC (%)**	**% mol FA in *sn*-2 position**	**Type of fat**	**% mol FA in *sn*-2 position**
Cocoa butter [29]	16:0	24	6.8	Salatrim	not available
	18:0	35	6.8		
	18:1	36	29.4		
	18:2	3	3.0		
Olive oil [29]	16:0	10	0.3	HOSO [115]	0.1
	18:0	2	-		0.1
	18:1	76	28.0		92.9
	18:2	10	4.7		6.8
Palm oil [29]	16:0	40	4.4	Betapol [120]	72.7
	18:0	4	-		6.9
	18:1	43	23.1		14.7
	18:2	11	6.1		3.6
Peanut oil [110]	16:0	9	0.7	Randomized peanut oil [110]	4.3
	18:0	3	tr		1.2
	18:1	58	28.5		18.3
	18:2	23	12.9		9.6
Cottonseed oil [99]	16:0	24	2.0	Randomized cottonseed oil [99]	8.3
	18:0	3	0.2		0.9
	18:1	18	7.2		6.0
	18:2	53	25.0		17.7
Tallow [105]	16:0	25	3.8	Randomized tallow [105]	8.5
	18:0	17	2.2		6.2
	18:1	40	19.7		13.1
	18:2	3	1.7		0.8
Lard [105]	16:0	21	21.3	Randomized lard [105]	7.6
	18:0	11	1.2		3.9
	18:1	39	4.9		12.7
	18:2	16	2.3		4.7
Fish oil [94]	10:0	-	-	Randomized fish oil [94]	40.9
	14:0	8.5	12.6		5.2
	16:0	18	22.3		10.6
	18:0	2.1	0.5		1.2
	18:1	12.6	7.4		7.3
	18:2	2.9	3.0		1.7
	20:5	7.6	9.2		4.4
	22:6	11.2	21.4		6.5

#### Applications of interesterified fats

Randomized fats have diverse applications, both in the food industry and in clinical applications [15,66]. Interesterified fats and oils differ from their native products in their melting and crystallisation properties and this often confers desired rhealogical properties to bakery and confectionary products [24,25].

In clinical applications, randomization provides energy-rich substrates for parenteral, enteral and infant feeding that are well-absorbed [15,16]. For instance, the optimal randomization of medium-chain fatty acids in TAG structures will upon digestion yield *sn*-1 and *sn*-3 fatty acids, which will be absorbed directly into portal circulation [67]. This application is used in the development of enteral products benefiting patients with fat malabsorption disorders [68]. Structured lipid emulsions used in parenteral feeding of catabolically stressed patients are said to promote whole body fat oxidation and nitrogen balance [69-72].

The specific positioning of C16:0 at the *sn*-2 position in human milk fat has a biological functionality, as fat absorption is enhanced [73,74]. Similarly infant formula products incorporate Betapol™, an interesterified fat with C16:0 in the *sn*-2 position, to optimize fat absorption and minimize calcium excretion [75]. In contrast, poorly absorbed fats can be designed to benefit weight loss, such as C18:0 randomized with short-chain fatty acids (C2:0 to C4:0). An example is the formulation of Salatrim™ or Caprenin™ containing randomized caprylic, capric and behenic acids [76-78].

### Absorption of interesterified fats

#### Digestibility of interesterified fats

Early studies on synthetic fats by Kritchevsky [18] in rabbits, contributed to the understanding that both TAG positional isomerism and fatty acid composition of TAG structure affect fat digestibility. Digestibility falls steeply with increasing concentrations of tristearin compared to tripalmitin even at low fat concentration [79]. In rats fed 10% of a target fatty acid or glucose, measuring the coefficient of fat digestibility in terms of fat excretion, shows fatty acids 4 to 10 carbon-chain length are absorbed 100% compared to 86%, 48%, 12% and 84% for C12:0, C16:0, C18:0 and C18:1 [80].

The ability of fats and fatty acid saturation/unsaturation to affect uptake during digestion has been examined. TAGs comprising unsaturated fatty acids are more digestible compared to free fatty acids for C18:1 (99% vs 73%) and C18:2 (97% vs 84%) but the reverse is true for C16:0 (22% vs 40%) and C18:0 (14% vs 24%) [81]. Mattson [82] showed that tristearin (SSS) is totally unabsorbed compared to almost total absorption of mono- or distearin-unsaturates, using fat blends or randomized fats from safflower and hydrogenated linseed oils.

SSS on its own is indigestible, but its digestibility improves as a mixed glyceride [82,83]. With a total fat content of 15%, digestibility was shown in a rat study to be 39% with tristearin:triolein at 2:1, compared to 69% for a ratio of 1:2 [83]. Of the predominant TAG molecular species, 73% of SOO was digested compared to 59% for SSO, suggesting preferential hydrolysis for C18:1 in the *sn*-2 position compared to C18:0. Bergsted et al. [84,85] in comparing SSS and triolein (OOO) content of lymph, mucosa and lumen of rats, found 65% less SSS in lymph, 46% less in mucosa, but 660% more in lumen, in contrast to significantly reduced OOO in the small intestine. The absorptive index was 94.3% and 56.7% for OOO and SSS respectively. However, mixing SSS with OOO or tripalmitin (PPP), considerably improved the lymph output in the order of SSS < SSS+PPP < SSS+OOO.

#### Influence on dietary cholesterol absorption

Feldman et al. [86] investigated cholesterol absorption in rats in relation to tristearin, triolein and mixed natural TAGs from safflower oil. Absorption rates were least with tristearin (~46%) compared to triolein (~70%) or safflower oil (~75%). Accumulation in lymph was lowest with tristearin but greatest with sterol synthesis. In another rat study [87] comparing trilaurin, trimyristin, tripalmitin and tristearin, weight gain and cholesterol absorption was lowest with tristearin, whilst lymph accumulation was in the order of trimyristin (72%) > trilaurin (58%) > tripalmitin (49%) > tristearin (42%). Imazumi et al. [88] fed hamsters with high oleic safflower oil (HOSO), co-randomized with trilaurin, trimyristin, tripalmitin or tristearin (yielding 50% targeted SFA with 30% C18:1 and 20% C18:2) with or without 2% cholesterol. Stearic acid-rich cholesterol-free diets were associated with the lowest fat digestibility, and greatest steroid excretion.

### Metabolic processing of interesterified fats

Early events in the metabolic processing of synthetic TAGs, may potentially have a bearing on the development of risk factors for coronary heart disease (CHD) [20,34,36,41]. Fundamental to this hypothesis would be our understanding that enzyme hydrolysis during digestion favors fatty acids in the sn-1/3 positions of TAGs in contrast to only 22% of fatty acids in the *sn*-2 position. Further, there is substantial preservation of the fatty acid at the *sn*-2 position of the ingested TAG in chylomicrons after digestion, and overall stereospecific structure of the ingested TAG is largely preserved in chylomicrons.

#### Animal studies

Studies by Innis et al [89,90] lend supportive evidence, that manipulation of beneficial *sn*-2 fatty acids in dietary TAGs, takes into account their conservation during digestion, absorption and reassembly into chylomicron-TAGs. Newborn piglets were fed sow's milk (55% *sn*-2 C16:0), or formulas based on synthesized fats (32% *sn*-2 C16:0), or palm olein (4.2% *sn*-2 C16:0) for 18 days from birth [89]. Measured *sn*-2 C16:0 in chylomicron-TAGs, was dose-dependent to the amount of *sn*-2 C16:0 in the following order: sow's milk > synthesized fats > palm olein. In comparing formula feeding in piglets, the positional distribution of C16:0 was the main determinant in weight gain per liter formula fed, which was greatest with C16:0 in the *sn*-2 position [90].

Lower concentrations of C20:4*n*-6 and C22:6*n*-3, in chylomicron-TAGs, were associated with synthetic fat feeding in the piglet study discussed earlier, compared to a formula with palm olein [90]. Positional differences in SFAs were hypothesized to modulate the transport of *n*-6 and *n*-3 PUFAs [89]. A rat study indicated that dietary fatty acids in the *sn*-2 position could influence lipemia and platelet reactivity, as well as desaturation and elongation of PUFAs [91]. The *sn*-2 positions of C18:2, C16:0 and C18:1 were directly related to plasma levels of C20:4*n*-6, C16:1*n*-7 and C20:3*n*-9 respectively.

#### Human studies

The natural prevalence of *sn*-2 16:0 (at 66%) in human milk fat supports the hypothesis for the enhanced absorption of *sn*-2 fatty acids in TAG molecules [73,74]. Altering the fatty acid at the *sn*-2 position of diet-TAGs affects the amount of fat absorbed, as shown in infants fed formula milk containing randomized lard (*sn*-1/3 16:0 > *sn*-2 16:0) *versus *native lard (*sn*-1/3 16:0 <*sn*-2 16:0) [74]. Fat excretion increased 6-fold for infants fed formula containing randomized lard, compared to native lard. The association of increased fat excretion with *sn*-1/3 16:0, was also confirmed in an infant-feeding trial with vegetable oils providing *sn*-2 16:0 at 19% (native) or 44.5% (randomized) [92]. In contrast to infants, adults are hypothesized to have a higher efficiency of absorption irrespective of positional distribution of fatty acids, but studies supporting this view are inconsistent [66].

According to Summers et al. [20] early metabolic events, caused by enzymatic-hydrolysis of chylomicron-TAG, are not influenced by the nature or the position of fatty acids within dietary TAGs. They found no preferential handling of either C18:1 or C18:0 randomized at the *sn*-2 position in systemic circulation, or in subcutaneous adipose tissue. TAG hydrolysis *in vivo *in adipose tissue by lipoprotein lipase (LpL) was found to be highly efficient, and the composition of non-esterified fatty acids released from adipose tissue, matched the composition of diet-TAGs. They further reported, that there was no release of the fatty acid from 2-monoacylyglycerol, from either adipose tissue or chylomicrons, nor were there selective fatty acid uptake or release from chylomicron TAG, related to the nature or the *sn*-2 position of fatty acids. Clearly LpL did not differentiate between *sn*-2 saturated or *sn*-2 unsaturated fatty acids.

Symmetry or asymmetry of TAG molecules may make a difference in early metabolic processing. Sanders et al. [47,48] found Salatrim™, a randomized C18:0-rich TAG, or an unrandomized fat such as cocoa butter resulted in different postprandial effects on lipids and FVII. Symmetrical C18:0-rich TAG with C18:1 in the *sn*-2 position appeared to be absorbed more rapidly (postprandial peaking at 4 h) compared to asymmetrical TAG with long chain-SFAs in the *sn*-2 position (postprandial peaking at 5 h). Again, asymmetry may perhaps explain the initial low activity of pancreatic lipase towards *n*-3 fatty acids specific to the *sn*-3 position. This would trigger a lag phase in the supply of *sn*-2 monoacylglycerols, which eventually delays the formation of TAG in intestinal mucosal cells [37].

#### Metabolic processing of long chain fatty acids

To overcome the problem of slow hydrolysis of *n*-3 PUFAs, structured fats have been developed through the enzymatic interesterification of marine *n*-3 PUFAs with MCFAs, on the principle that MCFAs occupying sn-1/3 positions get hydrolyzed earlier than LCFAs in the same position. Structured oils predominating in MLM species with decanoic acid (C10:0) mainly in the outer position, or LML with C20:5n-3 in the *sn*-1/3 positions, are examples of oils produced through this process. Rat studies show hydrolysis rates are 2–3 times faster for MLM-TAGs compared to LML-TAGs [93]. Alternately, randomized fats have also been produced through chemical interesterification of fish oil with C10:0. A rat study using randomized fish oil (RFO), MLM, and LML, with native fish oil (FO) as a control found differences in lymphatic transport [94]. Transport of total TAGs was greater with FO and RFO compared to the structured oils MLM or LML. Higher transport of C20:5n-3 in LML and MLM reflected greater concentrations in the structured oils (for LML 12.2 mol% and for MLM 34.6%) at *sn*-2 position compared to either the FO (9.2 mol%) or RFO (4.4 mol%). Rapid absorption of C20:5n-3 after MLM, than from LML, was attributed to faster hydrolysis with C10:0 in the *sn*-1/3 positions. This phenomenon has been also observed in thoracic duct-cannulated rats, showing increased absorption of C18:2*n*-6 in the *sn*-2 position of MLM-type structured TAGs, compared to a trilinolein structured fat [95].

Two oils with equal amounts of C18:1, but differing in TAG molecular species and minor fatty acid components, produced different TRL-TAG responses in the postprandial state [21,96]. Major TAG species typical of virgin olive oil are OOO and POO, whereas OOO is substantially higher in high oleic acid sunflower oil (HOSO). Metabolic processing of TAG-TRL, despite OOO-rich olive oil and HOSO diets indicated a decrease in OOO up to 20% content [21]. The *sn*-2 fatty acids were largely preserved in chylomicron-TAGs, but not in VLDL-TAGs in which POO became the major TAG after feeding C18:1-rich diets [96]. VLDL-TAGs also contained significantly less C18:2, whilst arachidonic acid increased significantly in the *sn*-2 position, after feeding olive oil compared to HOSO. Olive oil, in particular, promoted the presence of α-linolenic acid and docosahexaenoic acid in the *sn*-2 position of VLDL-TAGs, possibly through competitive inhibition of arachidonic acid utilization by n-3 PUFA [21,96]. OOL, which was a minor component in both dietary oils, became a major component in TRL, which is hypothesized to be a mechanism for supplying tissues with essential C18:2 [21].

### Effects of interesterified fats on fasting lipids

A substantial amount of research, evaluating stereospecificity of dietary fats, has focused on C16:0, and to a lesser extent C18:0, in the need to develop infant milk products that closely resemble human milk. This led to the conservation of C16:0 at the *sn*-2 position, and the maxim "retention and not excretion" for maximizing dietary TAG absorption. The porcine model is relevant to evaluating absorption, excretion and lipoprotein parameters, due to its short growth period (18 days). The rabbit model allows rapid tracking for atherogenicity. Because of this, there is paucity of information on the effects of the stereospecific changes of other SFAs, particularly lauric and myristic, on lipoprotein and lipid parameters [16].

#### C16:0 versus C18:1 in the *sn*-2 position

Palm oil, with C16:0 in the *sn*-1/3 positions, and Betapol™, an interesterified fat with C16:0 at the *sn*-2 position were fed to newborn piglets [97]. The *sn*-2 16:0 resulted in higher TC and HDL-C concentrations compared to C18:1 in the *sn*-2 position, by the 18^th ^day. This rise is similar to feeding sow's milk, which has 55% of C16:0 in the *sn*-2 position [91].

Newborn piglets developed higher plasma TC and TAG when fed sow's milk (55% *sn*-2 C16:0) compared to either palm olein (4.2% *sn*-2 C16:0), or synthesized fat (32% *sn*-2 C16:0), with or without added cholesterol [90]. TC and TAG concentrations were similar in the piglets fed either palm olein or synthesized fat. Obviously, the amount of C16:0 in the *sn*-2 position was critical to affecting TC and TAG levels. Similarly, *sn*-2 C16:0 in lard elicited higher plasma TAG values than the *sn*-1/3 16:0 in palm oil, fed to rats [92].

A peculiarity reported in rabbit studies, is that interesterified fats are associated with the development of artherogenesis, even without abnormalities in lipid and lipoprotein metabolism. When native cottonseed oil (2% *sn*-2 C16:0) was compared to the randomized version (8 to14%, *sn*-2 C16:0), atherogenicity increased in rabbits fed randomized cottonseed oil, without change in TC and TAG concentrations [98,99]. Kritchevsky et al. [100] hypothesize that enhanced absorption, combined with prolonged postprandial effects of *sn*-2 C16:0 brings about increased exposure of the aorta to C16:0 and subsequent increased fat deposition. However, these effects could not be reproduced in the hamster model [16].

In human infants, plasma TAG values were similarly increased when fed a formula containing randomized palm oil, with more C16:0 in the *sn*-2 position [101]. In contrast, when normocholesterolemic adults were fed margarine prepared from palm oil (18% *sn*-2 C16:0 and 82% *sn*-1/3 C16:0) or palm oil interesterified with sunflower oil (65% *sn*-2 C16:0 and 35% *sn*-1/3 C16:0), only men experienced significant increases in TC and LDL-C concentrations, but not women [23]. Another human study, also noted positional differences in C16:0 made no significant difference to blood lipid and lipoprotein levels [102]. Given these conflicting results, it appears that human infants and piglets experience raised lipid and lipoprotein levels in response to *sn*-2 C16:0, but in adult humans this effect is not conclusive.

#### C16:0 versus C18:0 in the *sn*-2 position

McGandy et al. [65] found C18:0 randomized in synthetic fat, raised blood cholesterol in human subjects in a similar manner to C16:0 and C14:0 in native fats. In contrast, other workers showed either feeding cocoa butter, which had C18:0 at *sn*-1/3 positions, or an interesterified fat blend which had an equitable distribution of C18:0 in all three positions, did not change blood cholesterol or TAG levels [103,104].

Native lard (>C18:2 and <C18:0) and native tallow (<C18:2 and >C18:0) have similar amounts of C16:0 (24%), albeit in different positional distributions (90% of *sn*-2 C16:0 for lard compared to 15% for tallow) [105]. Randomization achieves an equitable positional distribution of the various fatty acids. When such fats were fed to rabbits, TC was not significantly different between treatments, but the animals developed various degrees of atherogenicity according to the type of fat fed [105]. The atherogenic potential of the randomized fats were in the following order: native lard > randomized lard = randomized tallow > native tallow. In measured terms, the amount of *sn*-2 C16:0 as a percentage of total fat in the four test fats were 21.6%, 8.5%, 7.6% and 3.6% respectively. An inference can be drawn that C18:0, even in the *sn*-2 position, was not as atherogenic as C16:0 which increased atherogenesis in a dose-dependent manner.

#### C12:0 versus C14:0 versus C16:0 versus C18:0 in the *sn*-2 position

Normocholesterolemic subjects were fed a blend of common vegetable oils (coconut, palm oil, palm oil-stearin and low-*trans *partially hydrogenated rapeseed oil) or a randomized version [106]. The fatty acid composition of both diets was similar, but the positional distribution differed with more C14:0, C16:0 and C18:0 at the *sn*-2 position in the interesterified fat at the expense of C18:1 and C12:0. Blood lipids and lipoprotein parameters were not significantly different between treatments, even when fed at two different energy levels (4% and 8% of energy).

Corn oil interesterified with trilaurin, trimyristin, tripalmitin and tristearin, yielded oils with similar iodine value but differing in a *sn*-2 SFA. Kritchevsky and Tepper [107] fed rabbits with 6% by weight of such structured fats and 2% cholesterol, compared to native or randomized corn oil. Fats containing *sn*-2 SFAs were more atherogenic than native or randomized corn oil, and C16:0 was the most atherogenic of the compared *sn*-2 SFAs. However, TC levels did not differ between test diets possibly because of the substantial presence of C18:2. In feeding normocholesterolemic premenopausal women, Snook et al. [108] kept C18:2 minimal (3% energy) in diets comparing different synthetic fats (trimyristin *vs. *tripalmitin *vs. *tristearin at 14% energy). TC, LDL-C and cholesterol ester concentrations increased the most with C16:0, followed by C14:0, whilst remaining unchanged with C18:0. Effects produced by C16:0 and C14:0 were however not statistically different, which the researchers attributed to subject phenotype differences causing hyper- and hypo-responses, specific to either fatty acid.

#### Long-chain saturated fatty acid (LC-SFA) in the *sn*-2 position

Long chain-SFAs in native peanut oil are mostly in the *sn*-3 position [109]. In primates, randomized peanut oil with *sn*-2 LC-SFAs (C20:0-C24:0) induced lesser atherogenicity than native peanut oil [110]. A rabbit study, comparing native and randomized peanut oil with corn oil, also found native peanut oil induced the highest degree of atherosclerosis, despite unchanged TC levels [111]. The reduced atherogenicity associated with randomization was attributed to lower lectin levels by Kritchevsky et al. [112], which otherwise occur in higher concentrations in native peanut oil. However, Hayes [15] suggests location of LC-SFAs at the *sn*-3 position in native peanut oil could render the *sn*-2 PUFA inaccessible, and this plausibly promotes atherogenicity.

A mix of behenic acid (C22:0) at ~45% and capric and caprylic acids (8:0–10:0) at ~50%, was used in the formulation of Caprenin™. Randomization allows 95% of the TAG molecules to contain C22:0. In hypercholesterolemic men fed Caprenin™, after two baseline diets enriched with palm oil/palm kernel oil or butter, comparisons after six weeks showed significant reductions in HDL-C, HDL_2_-C and HDL_3_-C [113]. Increase in the TC/HDL-C ratio after the Caprenin™ diet, without changes in TC, LDL-C, triglycerides, apo B-100 or apo A-1, was noted. Most dietary fats have C18:1 in a *sn*-2 position as shown in Table 1. OOO is the predominant TAG species in olive oil (50%) or high oleic sunflower oil (HOSO) (65%). In healthy human subjects, with or without hyperlipidemia, feeding diets enriched with NCEP Step 1 diet (MUFA = 12% energy), or MUFA-enriched diets (+10% energy exchanged with carbohydrate energy) with either olive oil or HOSO, produced similar plasma lipid and lipoprotein profiles [114,115].

Structured fats such as Salatrim™ or Caprenin™, designed to enable weight loss, have being evaluated for their effect on blood cholesterol [76-78]. Salatrim™ had no effect on blood lipids and lipoprotein metabolism, but Caprenin™ was shown to be hypercholesterolemic in men [113,116,117]. The hypercholesterolemic effect of Caprenin™ was discussed previously. The different effects may be possibly attributed to differences in fatty acid composition, rather than structural differences. Salatrim™ is comprised of C18:0 randomized with short-chain fatty acids (C2:0 to C4:0), whilst Caprenin™ is formulated with caprylic, capric (C10:0) and behenic acids (C22:0) [76-78].

A postprandial study [118] compared butter with fat replacers (Simplesse™, Olestra™ and Passelli™) and potato as control, with or without modified sham feeding (MSF). Simplesse™ is protein-based, whilst Passelli™ is carbohydrate-based. Oral exposure by MSF contributed little to postprandial TAG rise. Fat clearance was slower with butter compared to the fat-replacers. Olestra™ even reduced the impact of the initial fat challenge below baseline values. Olestra™ possibly had an impact on lipids stored in the lacteals or chylomicrons as well as fat absorbed from oral stimulants [118].

### Effects of interesterified fats on the postprandial state

Recognizing the atherogenic potential of TRL, attempts are underway to define the ideal TAG profile which is least lipemic postprandially. The traditional long-term feeding approach, used in most dietary studies, is not optimal for characterizing fatty acid behavior, with respect to HDL-C, TAG and TRL generation. The postprandial model, on the other hand, offers a more valid approach in assessing the stereospecificity of fatty acids in relation to lipemia. In a definitive sense, postprandial lipemia represents exogenous lipids drawn from the diet. The degree and magnitude of postprandial lipemia, and the nature of TRLs in chylomicron, are critical to the metabolic processes which generate TRL remnants and small dense LDL [8]. Studies using the postprandial lipemic model started since the 1990s, and preferably utilized human subjects, either normocholesterolemic or with lipoprotein abnormalities. Information on the influence of dietary TAG structures on plasma TAG levels, therefore, contributes to an understanding of the atherogenic potential of TRL-TAGs.

#### C16:0-rich fats

Purified LpL from human and animal sources, display substrate specificity depending on fatty acid and TAG composition of the diet [53,54]. When fat-enriched chylomicrons or lipid emulsions were injected into rats, both hydrolysis of TAGs by LpL and liver uptake of remnants were slower with a SFA in the *sn*-2 position [119,120]. However, in a postprandial model with human subjects, Zampelas et al. [121] found TAG clearance, during lipemia, was not affected by changing the position of dietary C16:0 from *sn*-1/3 positions to the *sn*-2 position.

Myher et al. [122] found chylomicrons reflected considerably more *sn*-2 16:0 in humans fed lard, compared to a control diet with Cl6:0 distributed less at *sn*-2 and more at *sn*-1/3. With piglets, fed palm oil (4% *sn*-2 16:0) or sow's milk (55% *sn*-2 16:0) or formula containing interesterified palm, sunflower and canola oils (70% *sn*-2 16:0), incorporation of C16:0 into plasma TAG and cholesterol esters 4 h after feeding, was linearly dose-dependent to the amount of *sn*-2 16:0 fed [123]. A rat study showed more C16:0 was absorbed, with increased supply of *sn*-2 16:0 [124].

In comparing native palm oil and transesterified palm oil in a human postprandial study, Yli-Jokipii et al. [19,125] did not find a preferential absorption of *sn*-2 C16:0. They differentiated between dietary TAGs, chylomicron-TAGs and VLDL-TAGs arising from feeding native and randomized palm oil. For chylomicron-TAGs, similarities in the fatty acid composition between fat loads suggested C16:0, irrespective of positional distribution, was absorbed equally. Also, similarities in positional distribution of chylomicron-TAG and diet-TAG structures indicated the conservation of TAG structures relative to the test fat loads. In contrast, VLDL-TAG structures were different from the diet-TAGs, with endogenous synthesis as a possible source. The proportion of TAGs with two C18:1 residues and one C16:0 residue (POO+OOP and OPO), was reduced significantly in both chylomicrons and VLDL after both fat loads, and this lower production suggested endogenous TAG synthesis via glycerol-3-phosphate pathway. A key difference, arising from change in the positional distribution of C16:0 reported in their first study was VLDL-TAG structures were similar for both fat loads, in all but one regioisomer (POL+LOP and PLO+OLP) [19]. Additionally, the *sn*-1/3 16:0 fat caused a larger incremental area for plasma total TAGs, but reduced plasma insulin compared to the *sn*-2 16:0 fat. In the second study, Yli-Jokipii et al. [125] reported a slower rate of chylomicron TAG clearance for transesterified palm oil, compared to the native palm oil.

#### C18:1-rich fats

Virgin olive oil (VOO) and HOSO are two oils with equal amounts of C18:1, but with different compositions of minor fatty acids and TAG molecular species. VOO has a higher content of C16:0, C16:1n-7 and C18:3, whereas HOSO has higher proportions of C18:0 and C18:2. In a postprandial human study by Abia et al. [21], both oils elicited equal total TAG plasma concentrations, but their TAG-TRL responses during the postprandial period were significantly lowered by VOO, compared to HOSO. C18:1 content did not appear to influence postprandial metabolism, but rather minor fatty acids, C18:0 and C18:2 and their positional distribution in TAG had physiological relevance. Analysis of 2-monoacylglycerols demonstrated higher proportions of C18:0 and C16:0 and less C18:1 in TRL-TAGs derived from HOSO, compared to VOO TAG-TRL.

#### C18:0-rich fats

In a series of human postprandial studies, Sanders and co-workers examined the influence of the TAG structure in a variety of C18:0-rich fats on postprandial lipemia. A randomized C18:0-rich fat, obtained through the interesterification of fully hydrogenated with unhydrogenated sunflower oil (IE-SO), was shown to cause a lesser lipemia compared to C18:1-rich HOSO oil [47]. This study also showed reduced activated FVII concentrations with the randomized C18:0-rich fat, compared to the C18:1-rich HOSO. Conversely by using cocoa butter with 18:0 primarily in *sn*-1/3 positions, increased lipemia and activated FVII similar to the 18:1-rich HOSO, whereas Salatrim™, a synthetic randomized fat, produced a neutral effect [48]. Both randomized cocoa butter and IE-SO, which have more C18:0 in the *sn*-2 position, produced lower lipemia and lower activated FVII levels, compared to unrandomized cocoa butter [126,127].

Another native fat shea butter, despite having a high proportion of C18:0 in the sn-1/3 positions produced a lower lipemic response compared to C18:1-rich HOSO [128]. Lipemia was unaffected between native and randomized shea butter [22], while the *sn*-2 content of dietary TAGs was maintained in chylomicrons. The lipemic-lowering effects of randomized C18:0-rich fats were hypothesized to increase availability of C18:0 in the *sn*-2 position. Shea butter's ability in modulating this effect was also attributed to a lower C16:0 content compared to cocoa butter.

Alternately, randomized C18:0-rich fats having a high solid content at 37°C, could retard the absorption of fat [129]. Randomization increases the proportions of trisaturated TAG species, which would cause higher melting points compared to TAG species of increasing unsaturation [26]. However, Shahkhalili et al. [130] found no differences in the amount of fat absorbed and excreted, when testing randomized and unrandomized C18:0 rich fats. A likely explanation may also lie in the symmetry of dietary TAG molecules. Asymmetrical TAGs lead to activation of FVII, which could potentially increase thrombogenic risk [126]

#### Variable *sn*-2 fatty acids

Six interesterified fats were made up to ≈ 43% by weight by Tholstrup et al [131] with specific target fatty acids at the *sn*-2 position. Test fats were produced by interesterification of pure TAG (tristearin, tripalmitin, trimyristin) with high oleic-sunflower oil. Lipemic trends for all test fats peaked at 4 h. Stearic and palmitic acid rich fats caused lower lipemias, and slower return to the post-absorptive state, compared to MUFAs (both *cis *and *trans*) and PUFAs. Fatty acid chain length and degree of saturation influenced the extent and duration of lipemia, and hepatic VLDL output. LpL activity followed trends similar to that of lipemia. Cholestryl ester transfer protein (CETP) activity was not stimulated by most fatty acids during the prandial events, except for t*rans *18:1 which induced greater CETP activity compared to oleic acid after 4 h, A summary of human studies reviewed in this section is provided in Table 3.

**Table 3:** Effects of interesterified fats in the lipemic model

**Fat class**	**Reference**	**Study population**	**Study design**	**Diet**	**Endpoints**
Palm olein (*sn*-1/3 C16:0) *vs *Betapol™/interesterified palm olein (*sn*-2 C16:0)	Zampelas et al. [121]	16 healthy men Mean age: 24.8 ± 2.6 y Mean BMI: 22.7 ± 2.4 kg/m^2^	Randomized cross-over 2-wk wash-out 6 hr postprandial event Bleeds at -10, 0, 15, 30, 45, 60, 90, 120, 180, 240, 300, 360 min	Liquid meal: Meal replacement diet (Carnation Slender) + 40 g test fat + 200 ml water	Fat clearance: no difference Glucose, insulin and GIP: no difference

Palm olein (*sn*-1/3 C16:0) *vs *interesterified palm olein (*sn*-2 C16:0)	Yli-Jokipii et al. [19]	11 women age: 18–45 y BMI: 18.5–25 kg/m^2^	Double-blind Randomized cross-over 4-wk wash-out 6 hr postprandial event	Identical FAC with 32.8% of 16:0 Positional distribution: 16:0 in *sn*-1/3 for PO and in *sn*-2 for interesterified PO Liquid fat load-55 g/m^2 ^BSA	Fat clearance: no difference IAUC TAG: *sn*-1/3 C16:0 > *sn*-2 C16:0 Insulin: *sn*-1/3 C16:0 <*sn*-2 C16:0 Chylomicron-TAG structure: similar to diet TAG structure VLDL-TAG structure: similar for both test meals but dissimilar to diet origin.

Butter *vs *Fat replacers (Paselli™ vs Simplesse™ vs Olestra™)	Mattes [118]	2 men and 15 women Age: 28.5 ± 1.9 y BMI:26.1 ± 1.4 kg/m^2^	Randomized crossover 80 g almond evening meal prior to postprandial day Modified sham feeding (MSF) for oral stimulation 0 h: baseline bleed +10 min: 50 g safflower oil capsule + 20 min: blood drawn + every 3 min for 60 min: MSF + every 15 min for 60 min: MSF + bleeds at 2.5 h, 4.5 h, 6.5 h, 8.5 h after baseline	MSF @ 2 hr after baseline. Mashed potato alone as control. Test fat (= 50 g) fed in capsules with mashed potato.	AUC TAG: Butter > potato = Paselli™ = No MSF > Simplesse™ > Olestra Serum 18:1, apo B48, apo B 100: no treatment effects

Interesterified C18:0-rich sunflower oil *vs *C18:1-rich HOSO	Sanders et al. [47]	16 healthy subjects: 11 men, 5 women mean age (yr): 25.5 mean BMI (kg/m^2^): 23.2	Randomized crossover 7 hr postprandial events 0,1,2,4 and 5 hr drawn capillary blood 3 and 7 hr venous blood	Test fat : 90 g *vs *Low fat = 10 g Test meal challenge: cottage pie + banana milkshake with total nutrient value of 5.2 MJ, 37 g protein Fat-free lunch (1.7 MJ) after 3 hr	FVIIa: *sn*-2 C18:1 > *sn*-2 C18:0 Plasma TAG: *sn*-2 C18:1 > *sn*-2 C18:0

C18:0-rich cocoa butter (*sn*-1/3 C18:0) *vs *C18:1-rich HOSO (*sn*-2 C18:1) *vs *randomized C18:0-rich Salatrim™ (*sn*-2 C18:0)	Sanders et al. [48]	17 men and 18 women mean age (yr): men 51.3; women 46.2 mean BMI (kg/m^2^): men 25.9 and women 26.5	Randomized crossover 3 wk feeding per phase with 1 wk wash-out 0, 3 and 6 hr postprandial events	Test fat:30 g Test meal: muffin + strawberry milkshake Total nutrient value: 50 g fat, 17 g protein, 48 g carbohydrate	FVIIa: *sn*-1/3 C18:0 = *sn*-2 C18:1 > *sn*-2 C18:0 Plasma TAG: *sn*-1/3 C18:0 = *sn*-2 C18:1> *sn*-2 C18:0

C18:0 rich cocoa butter (*sn*-1/3 C18:0) *vs* interesterified cocoa butter (*sn*-2 C18:0)	Sanders et al. [126]	17 healthy males mean age: 38.2 yr mean BMI: 24.5 kg/m^2^	Randomized crossover 3 wk feeding per phase ≥ 1 wk wash-out 1,2,4 and 5 h postprandial events	Test meal: milkshake + muffin Nutrient value: 3.13 MJ, 16 g protein+ 50 g carbohydrate + 50 g test fat 3 h post-meal: fruit and low-fat yoghurt	FVIIa: *sn*-1/3 C18:0 <*sn*-2 C18:0 Plasma TAG: *sn*-1/3 C18:0 > *sn*-2 C18:0

C18:0 rich cocoa butter (*sn*-1/3 C18:0) *vs* Interesterified C18:0-rich sunflower oil (*sn*-2 C18:0)	Berry & Sanders [128]	6 healthy males age: 20–40 yr	Randomized crossover 6 hr postprandial event	Test fat: 50 g	FVIIa: *sn*-1/3 C18:0 <*sn*-2 C18:0 Plasma TAG: *sn*-1/3 C18:0 > *sn*-2 C18:0

Unrandomized shea butter (*sn*-1/3 C18:0) *vs *randomized shea butter (*sn*-2 C18:0)	Berry & Sanders [22]	16 healthy males	Randomized crossover trial	Test fat: 50 g Test meal: muffins	FVIIa: *sn*-1/3 C18:0 = *sn*-2 C18:0 Plasma TAG: *sn*-1/3 C18:0 = *sn*-2 C18:0

Unrandomized shea butter (*sn*-1/3 C18:0) *vs *C18:1-rich HOSO (*sn*-2 C18:1)	Berry & Sanders [129]	13 healthy males age: 20–43 yr	Randomized crossover trial 6 hr postprandial event	Test fat: 50 g	FVIIa: *sn*-2 C18:1 > *sn*-1/3 C18:0 Plasma TAG: *sn*-2 C18:1 > *sn*-1/3 C18:0

Interesterified fatty acids *sn*-2 C18:0 (n = 16) *sn*-2 C16:0 (n = 16) *sn*-2 C18:2 (n = 16) *sn*-2 C18:1 (n = 16) *sn*-2 C14:0+C16:0 (n = 8) *sn*-2 *trans *C18:1(n = 15)	Tholstrup et al. [131]	16 healthy men age: 21 to 28 yr BMI: 19.5 to 28.1 kg/m^2^	Crossover design ≥ 3 wk wash-out 2 days familiarization	Total fat energy-50.6% SFA:PUFA:MUFA = 40:41:19 Interesterified fatty acids targeted at ≈ 43% by wt Single meal fat-loading:1 g/kg BW	Fat clearance: C18:0 = C16:0 < C16:0+C14:0 <*cis *and *trans *C18:1 < C18:2 LpL: C18:0 = C16:0 = C16:0+C14:0 <*cis *C18:1 <*trans *C18:1 < C18:2 CETP: ↑ for *trans *C18:1 after 4 h; ↓ for C18:0 and C18:2 until 4 h; no change for the rest.

### Metabolic implications on altering stereospecificity of TAGs

Zock et al. [23] hypothesize fatty acids in the *sn*-2 position are preferentially transported to the liver instead of the extrahepatic tissues. This was based on the specificity of LpL in attacking the *sn*-1/3 positions of the TAG molecule [33,53]. In the liver, SFAs in the *sn*-2 position could preferentially affect LDL metabolism compared to the same SFA at the *sn*-1/3 positions [132].

Hayes [15] infers that potential benefit or harm to lipoprotein metabolism from structured fats, might originate from the consumption of specific TAG species related to aspects of TAG digestion, absorption and gut TAG resynthesis. The *sn*-2 monoacylglycerols, which are mostly PUFA or MUFA in native fats, are absorbed intact and serve as primary backbones for gut or liver phospholipid synthesis. Limiting PUFA availability, or insertion of a dysfunctional fatty acid such as C22:0 or *trans *18:1, is viewed by Hayes [15] as bearing potential harm to lipoprotein and eicosonoid metabolism. Displacing PUFA or MUFA from the critical *sn*-2 position, by substitution with a SFA or TFA, may directly alter lipoprotein metabolism through down-regulation of rLDL.

Alternately change in lipoprotein metabolism may be mediated through the placement of a unique fatty acid in the *sn*-2 position in the phospholipid structure [15]. Changes to phospholipid structure may affect downstream metabolic activities involving lecithin:cholesterol acyltransferase (LCAT) activity in the circulation. Unesterified cholesterol within circulating plasma lipoproteins is esterified to form cholesteryl esters (CE) by LCAT, which will then be transferred to HDL during reverse cholesterol transport. Further, acyl coenzyme A: cholesterol acyltransferase (ACAT) activity in the liver will promote the esterification of FC, shifting the equilibrium in favor of the CE pool, which leads to the 'up-regulation' of rLDL and reduced LDL-C levels [133]. Additionally, potential benefits or harm may be mediated through eicosonoid metabolism, such as the release of *sn*-2 fatty acids from phospholipids by phospholipase A2, or the generation of specific diacylglycerol from membrane phosphatidyl inositol by phospholipase C.

## Summary

Stereospecificity of most native oils and fats favor PUFA or MUFA in the *sn*-2 position, whilst SFAs are mainly distributed at the *sn*-1/3 positions. Interesterification has the capacity to invert this distribution by placing saturated fatty acids in the *sn*-2 position. Stereospecific structure and the *sn*-2 fatty acid position of the ingested TAG, are substantially preserved in chylomicrons. However, dietary TAG structure may not be conserved in VLDL-TAGs, as seen by the increase of POO after feeding C18:1-rich diets. Early metabolic events caused by LpL-hydrolysis of chylomicron-TAG are not influenced by the nature or the position of fatty acids within dietary TAG. Clearly LpL does not differentiate between *sn*-2 saturated or *sn*-2 unsaturated fatty acids. However, symmetry or asymmetry of TAG molecules may possibly make a difference in early metabolic processing, as symmetrical TAG molecules with C18:1 occupying the *sn*-2 position promotes a more rapid postprandial clearance from circulation.

Displacing PUFA or MUFA from the critical *sn*-2 position, by substitution with SFA, is hypothesized to cause lipid and lipoprotein abnormalities. Studies comparing the chain length of SFAs are limited, but indicate that saturates are most detrimental in the *sn*-2 position. For example, the amount of C16:0 in the *sn*-2 position influences TC and TAG levels in piglets and human infants. However, this effect has not been clearly demonstrated in human studies. In the rabbit model *sn*-2 16:0 appears to contribute to the development of atherogenicity in a dose-dependent manner, but other animal models do not show this effect. Overall, it is still too early to conclude on the behavior of unusual positional distribution of fatty acids in TAG species originating from dietary fats and oils. More studies that reflect postprandial events will elucidate the type of TAG molecular species in TAG-rich lipoproteins, that are either directly atherogenic, or influence the development of small and dense LDL particles.

## Declaration of Competing interests

No competing interests are declared. Financial support for this study was provided by the Malaysian Palm Oil Board, Malaysia, where Dr. Sundram was employed and Dr. T. Karupaiah was a graduate student. Both authors have read and approved the contents of this manuscript. The authors also dedicate this to the memory of Dr. David Kritchevsky, a friend and mentor, for his leadership in elucidating the impact of triacylglycerol structure on health and nutrition.

## References

[B1] Patsch JR, Miesenböck G, Hopferwieser T, Mühlberger V, Knapp E, Dunn JK, Gotto AM, Patsch W (1992). Relation of triglyceride metabolism and coronary artery disease. Studies in the postprandial state. Arterioscler Thromb.

[B2] Hokanson J, Austin MA (1996). Plasma triglyceride level is a risk factor for cardiovascular disease independent of high density lipoprotein cholesterol: a meta-analysis of population-based prospective studies. J Cardiovasc Risk.

[B3] Reardon MF, Nestel PJ, Craig IH, Harper RW (1985). Lipoprotein predictors of the severity of coronary artery disease in men and women. Circulation.

[B4] Hodis HN, Mack WJ, Azen SP, Alaupovic P, Pogoda JM, LaBree L, Hemphill LC, Kramsch DM, Blankenhorn DH (1994). Triglyceride- and cholesterol-rich lipoproteins have a differential effect on mild/moderate and severe lesion progression as assessed by quantitative coronary angiography in a controlled trial of lovastatin. Circulation.

[B5] Bolibar I, Thompson SG, von Eckardstein A, Sandkamp M, Assmann G (1995). Dose-dependent relationship of serum lipid measurements with the extent of coronary stenosis. Strong, independent, and comprehensive. Arterioscler Thromb.

[B6] Tkác I, Kimball BP, Lewis G, Uffelman K, Steiner G (1997). The severity of coronary atherosclerosis in type 2 diabetes mellitus is related to the number of circulating triglyceride-rich lipoprotein particles. Arterioscler Thromb.

[B7] Sharrett AR, Patsch W, Sorlie PD, Heiss G, Bond MG, Davis CE (1994). Association of lipoprotein cholesterols, apolipoprotein A-1 and B, and triglycerides with carotid atherosclerosis and coronary heart disease. Arterioscler Thromb.

[B8] Patsch JR (1994). Triglyceride rich lipoproteins and atherosclerosis. Arterosclerosis.

[B9] Roche HM, Gibney MJ (1997). Postprandial coagulation factor VII activity: the effects of monounsaturated fatty acids. Brit J Nutr.

[B10] Karpe F (1999). Postprandial lipoprotein metabolism and atherosclerosis. J Int Med.

[B11] Hennig B, Toborek M (2001). Nutrition and endothelial function: implications in atherosclerosis. Nutr Res.

[B12] Lechleitner M, Hoppichler F, Föger B, Patsch JR (1994). Low density lipoproteins of the postprandial state induce increased cellular cholesteryl ester accumulation in macrophages. Arterioscler Thromb.

[B13] Institute of Medicine (IOM) (2002). Letter report on dietary reference intakes for *trans *fatty acids. Dietary reference intakes for energy, carbohydrate, fiber, fat, fatty acids, cholesterol, protein and amino acids Institute of Medicine/Food and Nutrition Board.

[B14] Kubow S, Christophe AB (1998). The influence of stereospecific saturated fatty acids in dietary triacylglycerols on lipoprotein metabolism. Structurally modified food fats: synthesis, biochemistry, and use.

[B15] Hayes KC (2001). Synthetic and modified glycerides: effects on plasma lipids. Curr Opin Lipidol.

[B16] Hunter JE (2001). Studies on effects of dietary fatty acids as related to their position on triglycerides. Lipids.

[B17] Kritchevsky D (1988). Effects of triglyceride structure on lipid metabolism. Nutr Rev.

[B18] Kritchevsky D (1994). Stearic acid metabolism and atherogenesis: history. Am J Clin Nutr.

[B19] Yli-Jokipii K, Kallio H, Schwab U, Mykkänen H, Kurvinen J-P, Savolainen MJ, Tahvonen R (2001). Effects of palm oil and transesterified palm oil on chylomicron and VLDL triacylglycerol structures and postprandial lipid response. J Lipid Res.

[B20] Summers LKM, Fielding BA, Herd SL, Ilic V, Clark ML, Quinlan PT, Frayn KN (1999). Use of structured triacylglycerols containing predominantly stearic and oleic acids to probe early events in metabolic processing of dietary fat. J Lipid Res.

[B21] Abia R, Pacheco YM, Perona JS, Montero E, Muriana FJG, Ruiz-Gutiérrez V (2001). The metabolic availability of dietary triacylglycerols from two high oleic oils during the postprandial period does not depend on the amount of oleic acid ingested by healthy men. J Nutr.

[B22] Berry SEE, Sanders TAB (2005). Influence of triacylglycerol structure of stearic acid-rich fats on postprandial lipaemia. Proc Nutr Soc.

[B23] Zock PL, de Vries JHM, de Fouw NJ, Katan MB (1995). Positional distribution of fatty acids in dietary triglycerides: effects on fasting blood lipoprotein concentrations in humans. Am J Clin Nutr.

[B24] Padley FB, Gunstone FD, Harwood JL, Gunstone FD, Harwood JL, Padley FB (1994). Occurrence and characteristics of oils and fats. The Lipid Handbook.

[B25] Nawar WW, Fennema OR (1996). Lipids. Food Chemistry.

[B26] Small DM (1991). The effects of glyceride structure on fat absorption and metabolism. Ann Rev Nutr.

[B27] Brockerhoff H, Hoyle RJ, Wolmark N (1966). Positional distribution of fatty acids in triglycerides of animal depot fats. Biochim Biophys Acta.

[B28] Mattson FH, Lutton ES (1958). The specific distribution of fatty acids in the glycerides of animal and vegetable fats. J Biol Chem.

[B29] Brockerhoff H, Yurkowski M (1966). Stereospecific analyses of several vegetable fats. J Lipid Res.

[B30] Van der Wal RJ (1960). Calculation of the distribution of the saturated and unsaturated acyl groups in fats, from pancreatic lipase hydrolysis data. J Am Oil Chem Soc.

[B31] Martínez-Force E, Ruiz-López Ë, Garcés R (2004). The determination of the asymmetrical stereochemical distribution of fatty acids in triacylglycerols. Anal Biochem.

[B32] Decker EA (1996). The role of stereospecific saturated fatty acid positions on lipid metabolism. Nutr Rev.

[B33] Nillson-Ehle P, Egelrud T, Belfrage P, Olivecrona T, Borgstrom B (1973). Positional specificity of purified milk lipoprotein lipase. J Biol Chem.

[B34] Yang LY, Kuksis A (1991). Apparent convergence (at 2-monoacylglycerol level) of phosphatidic acid and 2-monoacylglycerol pathways of synthesis of chylomicron triacylglycerols. J Lipid Res.

[B35] Rogalska E, Ransac S, Verger R (1990). Stereoselectivity of lipases. II. Stereoselective hydrolysis of triglycerides by gastric and pancreatic lipases. J Biol Chem.

[B36] Mattson FH, Volpenhein RA (1964). The digestion and absorption of triglycerides. J Biol Chem.

[B37] Mu H, Høy C-K (2004). The digestion of dietary triacylglycerols. Prog in Lipid Res.

[B38] Schulthess G, Lipka G, Compassi S, Boffelli D, Weber FE, Paltauf F, Hauser H (1994). Absorption of monoacylglycerols by small intestinal brush border membrane. Biochemistry.

[B39] Stremmel W (1988). Uptake of fatty acids by jejunal mucosal cells is mediated by a fatty acid binding membrane protein. J Clin Investig.

[B40] Abumrad NA, Park JH, Park CR (1984). Permeation of long-chain fatty acids into adipocytes. J Biol Chem.

[B41] Pufal DA, Quinlan PT, Salter A (1995). Effect of dietary triacylglycerol structure on lipoprotein metabolism: a comparison of the effects of dioleoylpalmitoylglycerol in which palmitate is esterified to the 2-or 1(3)-position of the glycerol. Biochim Biophys Acta.

[B42] Kayden HJ, Senior JR, Mattson FH (1967). The monoglyceride pathway of fat absorption in man. J Clin Invest.

[B43] Tso P, Weidman SW, Horisberger M, Bracco U (1987). Absorption and metabolism of lipid in humans. Lipids in modern nutrition.

[B44] Bottino NR, Vandenburg GA, Reiser R (1967). Resistance of certain long-chain polyunsaturated fatty acids of marine oils to pancreatic lipase hydrolysis. Lipids.

[B45] Ikeda I, Sasaki E, Yasaunami H, Nomiyama S, Nakayama M, Sugano M, Imaizumi K, Yazawa K (1995). Digestion and lymphatic transport of eicosapentaenoic and docosahexaenoic acids given in the form of triacylglycerol, free acid and ethyl ester in rats. Biochim et Biophy Acta.

[B46] Christensen MS, Høy CE, Redgrave TG (1994). Lymphatic absorption of n-3 polyunsaturated fatty acids from marine oils with different intramolecular fatty acid distributions. Biochim et Biophy Acta.

[B47] Sanders TA, de Grassi T, Miller GJ, Morrissey JH (2000). Influence of fatty acid chain length and *cis/trans *isomerization on postprandial lipemia and factor VII in healthy subjects (postprandial lipids and factor VII). Atherosclerosis.

[B48] Sanders TA, Oakley FR, Cooper JA, Miller GJ (2001). Influence of a stearic acid-rich structured triacylglycerol on postprandial lipemia, factor VII concentrations, and fibrinolytic activity in healthy subjects. Am J Clin Nutr.

[B49] Hultin M, Olivecrona T (1998). Conversion of chylomicrons to remnants. Atherosclerosis.

[B50] Hultin M, Savonen R, Olivecrona T (1996). Chylomicron metabolism in rats: Lipolysis, recirculation of triglyceride-derived fatty acids in plasma FFA, and fate of core lipids as analyzed by compartmental modelling. J Lipid Res.

[B51] Park Y, Damron BD, Miles JM, Harris WS (2001). Measurement of human chylomicron triglyceride clearance with a labeled commercial lipid emulsion. Lipids.

[B52] Akesson B, Gronowitz S, Herslof B, Michelson P, Olivecrona T (1983). Stereospecificity of different lipases. Lipids.

[B53] Wang CH, Kuksis A, Manganaro F (1982). Studies of the substrate specificity of purified human milk lipoprotein lipase. Lipids.

[B54] Sato K, Suzuki K, Akiba Y (1998). Specific differences in substrate specificity of lipoprotein lipase purified from chickens and rats. Comp Biochem Physiol.

[B55] Bengtsson G, Olivercrona T (1980). Lipoprotein lipase: mechanism inhibition. Eur J Biochem.

[B56] Saxena U, Goldberg IJ (1990). Interaction of lipoprotein lipase with glycosaminoglycans and apolipoprotein C-II: effects of free fatty acids. Biochim Biophys Acta.

[B57] Saxena U, Witte LD, Goldberg IJ (1989). Release of endothelial cell lipoprotein lipase by plasma lipoproteins and free fatty acids. J Biol Chem.

[B58] Olivercrona T, Bengtsson-Olivercrona G (1990). Lipoprotein lipase and hepatic lipase. Curr Opin Lipidol.

[B59] Frayn KN, Shadid S, Hamlani R, Humphreys SM, Clark ML, Fielding BA, Boland O, Coppack SW (1994). Regulation of fatty acid movement in human adipose tissue in the postabsorptive-to-postprandial transition. Am J Physiol.

[B60] Rye K-A, Clay MA, Barter PJ (1999). Remodelling of high density lipoproteins by plasma factors. Atherosclerosis.

[B61] Redgrave TG (1970). Formation of cholesteryl ester-rich particulate lipid during metabolism of chylomicrons. J Clin Invest.

[B62] Quarfordt SH, Hanks J, Jones RS, Shelburne F (1980). The uptake of high density lipoprotein cholesteryl ester in the perfused rat liver. J Biol Chem.

[B63] Sherrill BC, Innerarity TL, Mahley RW (1980). Rapid hepatic clearance of the canine lipoproteins containing only the E apoprotein by high affinity receptor. J Biol Chem.

[B64] Mahley RW, Hui DY, Innerarity TL, Weisgraber KH (1981). Two independent lipoprotein receptors on hepatic membranes of the dog, swine, and man. Apo B, E and apo-E receptors. J Clin Invest.

[B65] McGandy RB, Hegsted DM, Myers ML (1970). Use of semisynthetic fats in determining effects of specific dietary fatty acids on serum lipids in man. Am J Clin Nutr.

[B66] Kubow S (1996). The influence of positional distribution of fatty acids in native, interesterified and structure-specific lipids on lipoprotein metabolism and atherogenesis. J Nutr Biochem.

[B67] Mu H, Porsgaard T (2005). The metabolism of structured triacylglycerols. Prog in Lipid Res.

[B68] Stein J (1999). Chemically defined structured lipids: current status and future directions in GI diseases. Internat J Colorectal Dis.

[B69] Pitkanen O, Takala J, Poyhonen M, Kari A (1991). Nitrogen and energy balance in septic and injured intensive care patients: response to parenteral nutrition. Clinical Nutr.

[B70] Sandstrom R, Hyltander A, Korner U, Lundholm K (1995). Structured triglycerides were well tolerated and induced increased whole body fat oxidation compared with long chain triglycerides in postoperative patients. J Paren Ent Nutr.

[B71] Kruimel JW, Naber TH, van der Vliet JA, Carneheim C, Katan MB, Jansen JB (2001). Parenteral structured triglyceride emulsion improves nitrogen balance and is cleared faster from the blood in moderately catabolic patients. J Paren Enter Nutr.

[B72] Lindgren BF, Ruokonen E, Magnusson-Borg K, Takala J (2001). Nitrogen sparing effect of structured triglycerides containing both medium and long chain fatty acids in critically ill patients: a double blind randomized controlled trial. Clinical Nutr.

[B73] Tomarelli RM, Meyer BJ, Weaber JR, Bernhart FW (1968). Effect of positional distribution on the absorption of the fatty acids of human milk and infant formulas. J Nutr.

[B74] Filer LJ, Mattson FH, Fomon SJ (1969). Triglyceride configuration and fat absorption by the human infant. J Nutr.

[B75] Lien EL, Boyle FG, Yuhas R, Tomarelli RM, Quinlan P (1997). The effect of triglyceride positional distribution on fatty acid absorption in rats. J Pediatr Gastroenterol Nutr.

[B76] Peters JC, Holcombe BN, Hiller LK, Webb DR (1991). Caprenin 3. Absorption and caloric value in adult humans. J Am Coll Toxicol.

[B77] Webb DR, Peters JC, Jandacek RJ, Fortier NE (1991). Caprenin 2. Short-term safety and metabolism in rats and hamsters. J Am Coll Toxicol.

[B78] Finley JW, Klemann LP, Leveille GA, Otterburn MS, Walchak CG (1994). Caloric availability of SALATRIM in rats and humans. J Agric Food Chem.

[B79] Hoagland R, Snider GG (1943). Digestibility of certain higher saturated fatty acids and triglycerides. J Nutr.

[B80] Carroll KK (1958). Digestibility of individual fatty acids in the rat. J Nutr.

[B81] Carroll KK, Richards JF (1958). Factors affecting digestibility of fatty acids in the rat. J Nutr.

[B82] Mattson FH (1959). The absorbability of stearic acid when fed as a simple or mixed triglyceride. J Nutr.

[B83] Mattil KF, Higgins JW (1945). The relationship of glyceride structure to fat digestibility. 1. Synthetic glycerides of stearic and oleic acids. J Nutr.

[B84] Bergstedt SE, Hayashi H, Kritchevsky D, Tso P (1990). A comparison of the absorption of glycerol tristearate and glycerol trioleate by rat small intestine. Am J Physiol.

[B85] Bergstedt SE, Bergstedt JL, Fujimoto K, Mansbach C, Kritchevsky D, Tso P (1991). Effects of glycerol tripalmitate and glycerol trioleate on intestinal absorption of glycerol tristearate. Am J Physiol.

[B86] Feldman EB, Russell BS, Schnare FH, Miles BC, Doyle EA, Moretti-Rojas I (1979). Effects of tristearin, triolein and safflower oil diets on cholesterol balance in rats. J Nutr.

[B87] Feldman EB, Russell BS, Schnare FH, Moretti-Rojas I, Miles BC, Doyle EA (1979). Effects of diets of homogenous saturated triglycerides on cholesterol balance in rats. J Nutr.

[B88] Imaizumi K, Abe K, Kuroiwa C, Sugano M (1993). Fat containing stearic acid increases fecal neutral steroid excretion and catabolism of low density lipoprotein without affecting plasma cholesterol concentrations in hamsters fed a cholesterol-containing diet. J Nutr.

[B89] Innis SM, Dyer R (1997). Dietary triacylglycerols with palmitic acid (16:0) in the 2-position increase 16:0 in the 2-position of plasma and chylomicron triacylglycerols, but reduce phospholipid arachidonic and docosahexaenoic acids and alter cholesteryl ester metabolism in formula-fed pigs. J Nutr.

[B90] Innis SM, Dyer R, Lien EL (1997). Formula containing randomized fats with palmitic acid (16:0) in the 2-position increases 16:0 in the 2-position of plasma and chylomicron triacylglycerols in formula-fed piglets to levels approaching those of piglets fed sow's milk. J Nutr.

[B91] Renaud SC, Ruf JC, Petithorny D (1995). The positional distribution of fatty acids in palm oil and lard influences their biologic effects in rats. J Nutr.

[B92] López-López A, Castellote-Bargalló AI, Campoy-Folgoso C, Rivero-Urgel M, Tormo-Carnicé R, Infante-Pina D, López-Sabater MC (2001). The influence of dietary palmitic acid triacylglyceride position on the fatty acid, calcium and magnesium contents of at term newborn faeces. Early Human Dev.

[B93] Nagata J, Kasai M, Watanabe S, Ikeda I, Saito M (2003). Effects of highly purified structured lipids containing medium-chain fatty acids and linoleic acid on lipid profiles in rats. Biosci Biotech & Biochem.

[B94] Porsgaard T, Mu H, Høy CE (2002). The influence of triacylglycerol structure on lymphatic transport, distribution between lipid classes, and synthesized triacylglycerol species in lymph after administration of structured lipids containing decanoic acid (10:0) and n-3 polyunsaturated fatty acids to rats.

[B95] Ikeda I, Tomari Y, Sugano M, Watanabe S, Nagata J (1991). Lymphatic absorption of structured glycerolipids containing medium-chain fatty acids and linoleic acid, and their effect on cholesterol absorption in rats. Lipids.

[B96] Ruiz-Gutiérrez V, Morgado N, Prada JL, Pérez-Jiménez F, Muriana FJG (1998). Composition of human VLDL triacylglycerols after ingestion of olive oil and high oleic sunflower oil. J Nutr.

[B97] Innis SM, Quinlan P, Diersen-Schade D (1993). Saturated fatty acid chain length and positional distribution in infant formula: effects on growth and plasma lipids and ketones in piglets. Am J Clin Nutr.

[B98] Kritchevsky D, Tepper SA, Wright S, Kuksis A, Hughes TA (1998). Cholesterol vehicle in experimental atherosclerosis. 20. Cottonseed oil and randomized cottonseed oil. Nutr Res.

[B99] Kritchevsky D, Tepper SA, Kuksis A, Wright S, Czarnecki SK (2000). Cholesterol vehicle in experimental atherosclerosis. 22. Refined, bleached, deodorized RBD palm oil, randomized palm oil and red palm oil. Nutr Res.

[B100] Kritchevsky D, Tepper SA, Chen SC, Meijer GW, Krauss RM (2000). Cholesterol vehicle in experimental atherosclerosis. 23. Effects of specific synthetic triglycerides. Lipids.

[B101] Carnielli VP, Luijendijk IHT, van Beek RHT, Boerma GJM, Degenhart HJ, Sauer PJJ (1995). Effect of dietary triacylglycerol fatty acid positional distribution on plasma lipid classes and their fatty acid composition in preterm infants. Am J Clin Nutr.

[B102] Nestel PJ, Noakes M, Belling GB, McArthur R, Clifton PM (1995). Effect on plasma lipids of interesterifying a mix of edible oils. Am J Clin Nutr.

[B103] Hegsted DM, McGandy RB, Myers ML, Stare FJ (1965). Quantitative effects of dietary fat on serum cholesterol in man. Am J Clin Nutr.

[B104] Grande F, Anderson JT, Keys A (1970). Comparison of effects of palmitic and stearic acids in the diet on serum cholesterol in man. Am J Clin Nutr.

[B105] Kritchevsky D, Tepper SA, Kuksis A, Eghtedary K, Klurfeld DM (1998). Cholesterol vehicle in experimental atherosclerosis. 21. Native and randomized lard and tallow. J Nutr Biochem.

[B106] Meijer GW, Westrate JA (1997). Interesterification of fats in margarine: effect on blood lipids, blood enzymes, and hemostatsis parameters. Euro J Clin Nutr.

[B107] Kritchevsky D, Tepper SA (1967). Cholesterol vehicle in experimental atherosclerosis X. Influence of specific saturated fatty acids. Exp Molec Pathol.

[B108] Snook JT, Park S, Williams G, Tsai Y-H, Lee N (1999). Effect of synthetic triglycerides of myristic, palmitic and stearic acid on serum lipoprotein metabolism. Euro J Clin Nutr.

[B109] Myher JJ, Marai L, Kuksis A, Kritchevsky D (1977). Acylgycerol structure of peanut oils of different atherogenic potentials. Lipids.

[B110] Kritchevsky D, Davidson LM, Weight M, Kriek NPJ, du Plessis JP (1982). Influence of native and randomized peanut oil on lipid metabolism and aortic sudanophilia in the vervet monkey. Atherosclerosis.

[B111] Kritchevsky D, Tepper SA, Vesselinovitch D, Wissler RW (1973). Cholesterol vehicle in experimental atherosclerosis Part 13. Randomized peanut oil. Atherosclerosis.

[B112] Kritchevsky D, Tepper SA, Klurfeld DM (1998). Lectin may contribute to the atherogenicity of peanut oil. Lipids.

[B113] Wardlaw GM, Snook JT, Park SM, Patel PK, Pendley FC, Lee MS, Jandacek RJ (1995). Relative effects on serum lipids and apolipoproteins of a caprenin-rich diet compared with diets rich in palm oil/palm-kernel oil or butter. Am J Clin Nutr.

[B114] Pérez-Jiménez F, Espino A, López-Segura F, Blanco J, Ruiz-Gutiérrez V, Prada JL, López-Miranda J, Jiménez-Perepérez J, Ordovas JM (1995). Lipoprotein concentrations in normolipidemic males consuming oleic acid-rich diets from two different sources: olive oil and oleic acid-enriched sunflower oil. Am J Clin Nutr.

[B115] Ruiz-Gutiérrez V, Muriana FJG, Guerrero A, Cert AM, Villar J (1996). Plasma lipids, erythrocyte membrane lipids and blood pressure of hypertensive women after ingestion of dietary oleic acid from two different sources. J Hypertens.

[B116] Auerbach MH, Chang PW, Kosmark R, O'Neill JJ, Philips JC, Christophe AB (1998). Salatrim: A family of reduced-calorie structured lipids. Structurally modified food fats: synthesis, biochemistry, and use.

[B117] Nestel PJ, Pomeroy S, Kay S, Sasahara T, Yamashita T (1998). Effect of a stearic acid-rich, structured triacylglycerol on plasma lipid concentrations. Am J Clin Nutr.

[B118] Mattes RD (2001). Oral exposure to butter, but not fat replacers elevates postprandial triacylglycerol concentrations in humans. J Nutr.

[B119] Mortimer B-C, Simmonds WJ, Joll CA, Stick RV, Redgrave TG (1988). Regulation of the metabolism of the lipid emulsion model lipoproteins by a saturated acyl chain at the 2-position of triacylglycerol. J Lipid Res.

[B120] Redgrave TG, Kodali DR, Small DM (1988). The effect of triacyl-sn-glycerol structure on the metabolism of chylomicrons and triacylglycerol-rich emulsions in the rat. J Biol Chem.

[B121] Zampelas A, Williams CM, Morgan LM, Wright J, Quinlan PT (1994). The effect of triacylglycerol fatty acid positional distribution on postprandial plasma metabolite and hormone responses in normal adult men. Br J Nutr.

[B122] Myher JJ, Kuksis A, Breckenridge WC, McGuire V, Little JA (1985). Comparative studies of triacylglycerol structure of very low density lipoproteins and chylomicrons of normolipemic subjects and patients with type II hyperlipoproteinemia. Lipids.

[B123] Innis SM, Dyer R, Quinlan P, Diersen-Schade D (1995). Palmitic acid is absorbed as *sn*-2 monopalmitin from milk and formula with rearranged triacylglycerols and results in increased plasma triglyceride *sn*-2 and cholesteryl ester palmitate in piglets. J Nutr.

[B124] Lien EL, Yuhas RJ, Boyle RG, Tomarelli RM (1993). Corandomisation of fats improves absorption in rats. J Nutr.

[B125] Yli-Jokipii KM, Schwab US, Tahvonen RL, Kurvinen J-P, Mykkänen HM, Kallio HPT (2002). Triacylglycerol molecular weight and to a lesser extent, fatty acid positional distribution, affect chylomicron triacylglycerol composition in women. J Nutr.

[B126] Sanders TAB, Berry SEE, Miller GJ (2003). Influence of triacylglycerol structure on the postprandial response of factor VII to stearic acid-rich fats. Am J Clin Nutr.

[B127] Sanders TAB, Oakley FR, Crook D, Cooper JA, Miller GJ (2003). High intakes of trans monounsaturated fatty acids taken for 2 weeks do not influence procoagulant and fibrinolytic risk markers for CHD in young healthy men. Brit J Nutr.

[B128] Berry SEE, Sanders TAB (2003). Postprandial lipaemia induced by cocoa butter compared with an inter-esterified blend of totally hydrogenated and hydrogenated high oleic sunflower oil. Proc Nutr Soc.

[B129] Berry SEE, Sanders TAB (2003). Physical properties of stearic acid-rich triacylglycerols modulate effects on postprandial lipaemia. Proc Nutr Soc.

[B130] Shahkhalili Y, Duruz E, Acheson K (2000). Digestibility of cocoa butter from chocolate in humans: a comparison with corn-oil. Euro J Clin Nutr.

[B131] Tholstrup T, Sandström B, Bysted A, Hølmer G (2001). Effect of 6 dietary fatty acids on the postprandial lipid profile, plasma fatty acids, lipoprotein lipase, and cholesterol ester transfer activities in healthy young men. Am J Clin Nutr.

[B132] Wang CH, Kuksis A, Manganaro F (1982). Studies of the substrate specificity of purified human milk lipoprotein lipase. Lipids.

[B133] Spady DK, Woollett LA, Dietschy JM (1993). Regulation of plasma LDL-cholesterol levels by dietary cholesterol and fatty acids. Annu Rev Nutr.

